# Preventing premature cardiovascular mortality: the role of lifestyle interventions and pharmacotherapy—a narrative review

**DOI:** 10.3389/fcvm.2025.1664802

**Published:** 2025-12-08

**Authors:** Marc Haber, Michael S. Nasr, Hadi Al Etri, Samer Nasr

**Affiliations:** 1 Faculty of Medicine and Medical Center, American University of Beirut, Beirut, Lebanon; 2 Faculty of Medicine, Lebanese American University, Beirut, Lebanon; 3 Division of Cardiology, Chairman of Medicine, University of Balamand, Beirut, Lebanon; 4 Department of Cardiology, Head of Cardiology, Mount Lebanon Hospital, Hazmieh, Lebanon

**Keywords:** cardiovascular disease prevention, premature mortality, lifestyle interventions, pharmacotherapy, risk stratification

## Abstract

Cardiovascular diseases (CVD), including coronary heart disease, cerebrovascular disease, and peripheral artery diseases, are the leading global cause of premature mortality in adults. Addressing CVD aligns with the United Nations Sustainable Development Goals aiming to reduce premature deaths by one-third by 2030 through lifestyle and pharmacological interventions. Major risk factors for CVD are categorized into lifestyle and genetic factors. Lifestyle factors such as elevated cholesterol, hypertension, high body mass index, smoking, poor dietary habits, and physical inactivity significantly increase CVD mortality. Conversely, genetic predisposition strongly influences individual risk, often amplified by unhealthy behaviors. Primary prevention strategies, including adherence to Dietary Approaches to Stop Hypertension (DASH) and Mediterranean Diet (MD), regular physical activity, and smoking cessation, have demonstrated effectiveness in reducing CVD incidence and mortality. Secondary prevention emphasizes pharmacological interventions, specifically aspirin and statin therapies, non-statin agents, antihypertensive, all of which significantly decrease recurrent cardiovascular events among high-risk individuals. Although cardiovascular screening practices remain debated, targeted screening informed by precision medicine approaches and artificial intelligence shows promise in stratifying risk effectively. This review synthesizes evidence on these preventive strategies, underscoring their integrated role in reducing premature CVD-related mortality, while recognizing the need for further implementation research to optimize preventive healthcare outcomes.

## 1 Introduction

**Figure 1:**
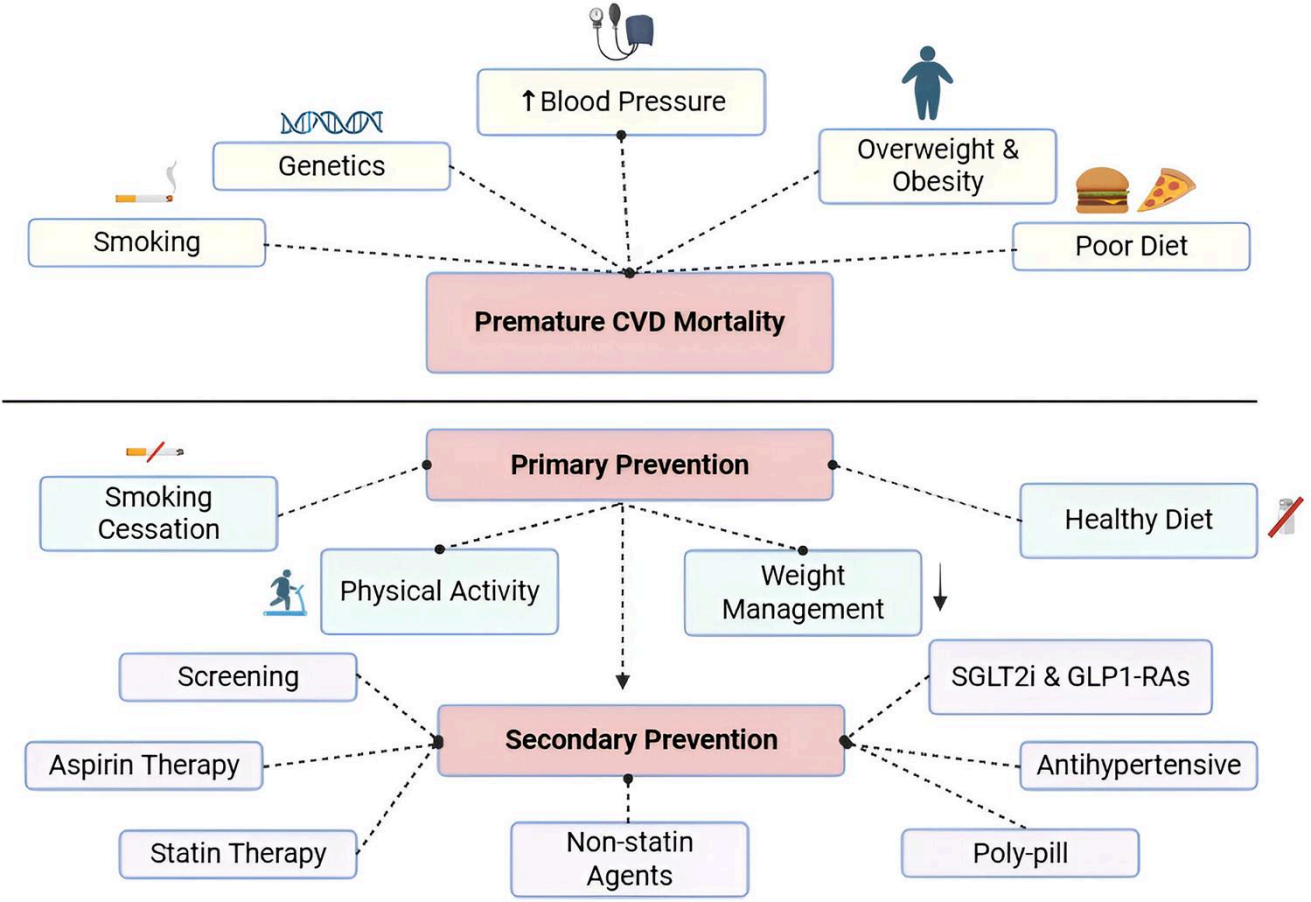
Summary of risk factors and prevention strategies for premature CVD mortality. Illustrations created with BioRender.com.

Cardiovascular diseases (CVD) are, as the name indicates, a group of disorders that affect the heart and blood vessels and include coronary heart diseases, cerebrovascular diseases, peripheral artery diseases, pulmonary embolism, and deep vein thrombosis (1). Heart diseases are the leading cause of death in the U.S (2). and globally (3) and are estimated to contribute 28% of premature deaths (1). The United Nations Sustainable Development Goals (SDGs) aim to reduce premature deaths by one third by the year 2030, including premature deaths from CVD (4). Some of the interventions listed under this indicator for SDGs include lifestyle modifications as well as pharmacological interventions (4). The American Heart Association's guidelines on the prevention of CVD focuses on lifestyle modifications (diet, physical activity, smoking) as the foundation of prevention. They also provide guidance on selective use of aspirin in CVD prevention, with recent updates highlighting the role of specific medications in the management of diabetes mellitus, a risk factor of CVD (5, 6). This review focuses on the evidence for dietary modifications, physical activity, smoking cessation, and pharmacological interventions in reducing premature CVD mortality, while also incorporating novel perspectives such as the role of artificial intelligence (AI) in risk prediction and emerging pharmacological therapies within the continuum of prevention (Table 1). Table 2 summarizes the studies described and Figure 1 presents a diagram of the risk factors and prevention strategies discussed in this review.

**Table 1:** Absolute effects (ARR) and NNT/NNH of Key pharmacological preventive strategies.

Author, Year Journal	Design & population	Follow-up duration	Intervention	Outcome	Absolute risk reduction Number needed to treat Number needed to harm
Baigent C, 2009 The Lancet	Meta-analysis 112,000	–	Aspirin	Prevention of serious vascular events	ARR: 0.06% (primary), 1.5% (secondary) NNT: 1,667 per year (Primary); 67 per year (Secondary) NNH: Major extracranial bleeds: 3,300 per year (Primary); 2,500 per year (Secondary)
ASCEND Study Group, 2018 NEJM	RCT 15,480	7.4 years	Aspirin	Prevention of major cardiovascular event	ARR: 1.1% NNT: 91 NNH: Major Bleeding Event: 112
Cannon C, 2015 NEJM	RCT 18,144	6 years	Ezetimibe (with and without aspirin)	Cardiovascular Morbidity and Death	ARR: 2.0% NNT: 50 NNH: Not significant
Sabatine M, 2017 NEJM	RCT 27,564	2.2 years	PCSK9i (evolocumab)	Cardiovascular Morbidity and Death	ARR: 1.5% NNT: 67 NNH: Injection-site reaction: 200
Yusuf S, 2021 Randomized Controlled Trials	RCT 5,713	4.6 years	Polypill with and without aspirin	CVD Risk	ARR: 1.1% (without aspirin); 1.7% (with aspirin) NNT: 91 (without aspirin); 59 (with aspirin) NNH: 67 (without aspirin); 63 (with aspirin)

**Table 2:** Summary of studies addressing different prevention strategies for premature CVD incidence and mortality.

Author Year Journal	Study design	Population	Intervention/exposure	Outcome	Results
Risk Factors					
Said Ma, 2019 Current Cardiology Report	Review	–	Lifestyle and Genetic Factors	CAD Events	There is interaction between genetics and lifestyle factors in CAD development
Kromhout D, 2007 The Lancet	Systematic review	900,000	Cholesterol Level	Vascular Mortality	Lower cholesterol levels are associated with lower vascular mortality
Lewington S, 2002 The Lancet	Systematic review	958,074	SBP and DBP	Vascular Mortality	Lower SBP and DBP is associated with decreased vascular mortality
Ettehad DM, 2016 The Lancet	Systematic review	613,815	Blood Pressure	CVD Event and Mortality	Lower BP is associated with decreased vascular risk
Di Angelantonio E, 2016 The Lancet	Systematic review	10,625,411	BMI	All-cause Mortality	Higher BMI was associated with higher all-cause mortality
Whitlock G, 2009 The Lancet	Systematic review	894,576	BMI	Cause-specific Mortality	Higher BMI was associated with higher vascular mortality
Bolijn R, 2023 Preventive Medicine Report	Cohort	18,058	Smoking	CVD Incidence	Smoking increased the incidence of CVD
Abu Jad AA, 2022 Cureus	Systematic reviews	8,000,000	Smoking	Tobacco and Cannabinoids impact of cardiovascular system	Cannabinoids had a higher negative impact on the cardiovascular system compared to tobacco
Chomistek AKS, 2015 JACC	Cohort	88,940	Lifestyle Factors	CVD cases and risk factors	Higher adherence to a healthy lifestyle decreased the risk of CVD development
Khera AV, 2016 NEJM	Systematic Review	55.685	Lifestyle and Genetic Factors	Coronary Events	Healthy lifestyle interaction with genetic risk decreased the coronary events
Diet					
Chiavaroli L, 2019 Nutrients	Umbrella of systematic reviews	946,554	DASH Diet	CVD Incidence	Following a DASH significantly decreased CVD incidence, SBP, and DBP
Schwingshackl LM, 2015 Nutrition and Dietetics	Systematic review	1,020,642	DASH Diet	All-cause Mortality. Cardiovascular Mortality and incidence	The DASH diet was associated with a decrease in all-cause mortality, cardiovascular mortality and incidence.
Cowell OR, 2021 Journal of Hypertension	Systematic review	63,138	Mediterranean Diet	Blood Pressure	The Mediterranean diet decreased SBP and DBP
Lotfi K, 2021 Advances in Nutrition	Systematic review	244,678	Mediterranean Diet	Overweight, Obesity, and Weight Change	There is a positive dose-response with adherence to Mediterranean diet and decrease in obesity/overweight risk
Liyanage T, 2016 PLOS ONE	Systematic review	10,950	Mediterranean Diet	Cardiovascular Outcomes	The Mediterranean diet was associated with a decrease in major cardiovascular events
Guo Y., 2025 Frontiers	Systematic review	10,962	Dietary Fibers Intake	Cardiovascular Mortality	Higher fiber intake was linked to lower all-cause and cardiovascular mortality, with evidence suggesting a threshold effect around moderate daily intake.
Mozaffarian D., 2010 PloS Medicine	Meta-analysis	13,614	Polyunsaturated fats replacing saturated fats	CHD Risk	Replacing saturated fats with polyunsaturated fats significantly reduced the risk of coronary heart disease in randomized controlled trials
Makarewicz, 2018 Public Health Nutrition	Systematic review	–	Saturated and trans-fatty acids replacement	CVD Risk	Recent large cohort studies and meta-analyses on saturated fat and cardiovascular risk have produced inconsistent findings
Mendoza K., 2024 The Lancet	Systematic review	–	Ultra-processed Foods	CVD Risk	Higher intake of ultra-processed foods was associated with increased risks of cardiovascular disease and coronary heart disease, with the strongest evidence for CHD
Wang Y., 2022 Nutrients	Systematic review	–	Sugar Sweetened Beverages Intake	Risk of Stroke	Higher consumption of sugar-sweetened beverages was associated with an increased risk of coronary heart disease–related outcomes, including ischemic stroke and cardiovascular mortality, with risks rising in a dose-dependent manner.
Smoking					
Piepoli MF, 2016 European Heart Journal	Guidelines	–	–	CVD Prevention	CVD is the underlying cause for 30% of CVD mortality
Saz-Lara A, 2021 European Journal of Cardiovascular Nursing	Systematic review	–	Smoking Cessation	Arterial Stiffness	Smoking cessation decreases arterial stiffness
Mons U, 2015, BMJ	Meta-analysis	503,905	Smoking Cessation	Cardiovascular event and mortality	There was a negative dose-response in CVD mortality with longer periods of smoking cessation
Duncan MS, 2019 Journal of the American Association	Cohort	8,770	Smoking Cessation	CVD Risk	Smoking cessation decreased the risk of CVD
Banks E., 2019 BMC Medicine	Cohort	188,167	Tobacco Smoking	CVD Risk	Smoking markedly increased the risk of major cardiovascular diseases and mortality in a dose-dependent manner, while quitting, especially before age 45, substantially reduced excess risk
Chen C., 2024 Addict Behaviors	Meta-analysis	8,499,444	Exclusive and dual use of combustible cigarettes	CVD Risk	Dual use of e-cigarettes and combustible cigarettes was strongly associated with higher cardiovascular risk
Jeffers A., 2024 JAHA	Population based cross sectional	434,104	Cannabis Use	CVD Risk	Daily cannabis use was associated with increased risks of myocardial infarction, stroke, and composite cardiovascular outcomes, with stronger associations observed among never-tobacco smokers and younger adults
Physical Activity					
Grassier B, 2021 Frontiers in Physiology	Systematic review	843	Training	Cardiovascular Health and Risk Factors	Training was associated with improved cardiovascular health and risk factors
Momma H, 2022 British Journal of Sports Medicine	Systematic review	263,058	Muscle-Strengthening	Major non-communicable risk and mortality	There was a negative dose-response with increased physical activity and a decrease in the risk of all-cause mortality and CVD mortality
Aune D, 2021 European Journal of Epidemiology	Systematic review	–	Physical Activity	Heart Failure	Total physical activity, leisure physical activity, vigorous physical activity, and walking physical activity were all associated with a decrease in heart failure
Obesity and Weight Management					
Riaz H, 2018 JAMA	Systematic review	881,692	Obesity	CAD Risk	Obesity was consistently linked to a higher risk coronary artery disease
Qiu Z, 2025 International Journal of Obesity	Systematic review	2,375,020	Obesity and BMI	CVD Risk	Higher BMI in early life was associated with increased risks of cardiovascular disease, coronary heart disease, and heart failure but not stroke, with stronger effects observed when BMI was measured in early adulthood
Chen X, 2025 International Journal of Obesity	Umbrella review	–	Weight Management	CVD Risk	GLP1-RAs, bariatric surgery, specific dietary patterns, and physical activity were associated with reduced cardiovascular risk
Screening					
Chen X, 2018 JAMA	Systematic review	77,140	Screening	Mortality and CVD events	Not enough evidence to screen
Bloetzer C, 2015 Public Health Reviews	Review	–	Screening	CVD Risk	Recommended screening for men and women aged 35 and 45. Not recommended to screen for CVD for these cut-off ages for men and women. Not recommended to screen for lipids in children to up to 20 years old
Singh M, 2024 The Lancet	Scoping Review	-	Screening	Capabilities of AI in CVD screening	AI improves CVD risk prediction and personalized care.
Medication					
Elwood PC, 1974 BMI	RCT	1,239	Aspirin	Myocardial Infarction Mortality	Aspirin reduced the risk of MI mortality
Baigent C, 2009 The Lancet	Meta-analysis	112,000	Aspirin	Prevention of vascular disease	Aspirin decreased the number of serious vascular events and the number of strokes
ASCEND Study Group, 2018 NEJM	RCT	15,480	Aspirin	Prevention of major cardiovascular event	Aspirin use led to a significant reduction in serious vascular events compared to placebo.
Herringtonn WG, 2016 The Lancet	Meta-analysis	183,419	Statin	LDL Cholesterol Level	Statin reduced the risk of major vascular event by lowering LDL cholesterol levels
Sultan S, 2023 Current Problems in Cardiology	Systematic review	31,018	Statin	CVD Prevention	Statin reduced the risk of major coronary and major cerebrovascular events
Sabatine M., 2017’ NEJM	RCT	27,564	PCSK9i (evolocumab)	CVD Risk	PCSK9i when combined with statin lowered LDL levels, in addition to a decrease in CVD risk
Cannon C., 2015 NEJM	RCT	18,144	Ezetimibe	Acute Coronary Syndrome	Ezetimibe when combined with statin lead to an incremental lowering of LDL levels, in addition to a decrease in CVD risk
Wang W, 2023 Mayo Clinic Proceedings	Systematic review	313,396	SGLT2i	Reduced CVD Risk	SGLT2 inhibitor use was associated with a substantially lower risk of cardiovascular disease compared with other second-line therapies
Usman M, 2023 Journal of the American College of Cardiology	Meta-analysis	90,413	SGLT2i	CVD Risk	SGLT2 inhibitors consistently reduced the risk of heart failure hospitalization and cardiovascular death across patients with heart failure
Rivera F, 2024 American Journal of Preventive Cardiology	Meta-analysis	83,258	GLP-1 RA	CVD Risk	GLP-1 receptor agonists significantly reduced major adverse cardiovascular events, cardiovascular and all-cause mortality, stroke, coronary revascularization, and kidney outcomes, with consistent benefits across sex, CVD history, BMI, and kidney function
Yusuf S., 2021 Randomized Controlled Trials	RCT	5,713	Polypill with and without aspirin	CVD risk	The polypill, especially when combined with aspirin, reduced cardiovascular events compared with placebo

BMI, body mass index; SBP, systolic blood pressure; DBP, diastolic blood pressure; CVD, cardiovascular disease; CAD, coronary artery disease; GLP1-RAs, glucagon-like peptide-1 receptor agonists; SGLT2i, sodium-glucose cotransporter-2 inhibitors; LDL, low-density lipoproteins; MI, myocardial infarction; AI, artificial intelligence.

## 2 Search strategy and selection criteria

The studies included were identified through searches in multiple databases, including PubMed, Medline, Embase, and Google Scholar, covering publications up to June 2025. Search terms combined cardiovascular disease, premature mortality, prevention, lifestyle interventions, and pharmacological therapies. Higher-level evidence was prioritized, giving preference to systematic reviews and meta-analyses, followed by randomized controlled trials and large prospective cohort studies. Only articles published in English and involving human populations were considered. Final inclusion of studies was determined by consensus among the authors.

## 3 Risk factors of CVD

Prior to discussing prevention strategies, it is important to consider the different risk factors of CVD which have been extensively studied in the last few decades. The most common risk factors can be lumped into two main categories: lifestyle factors and genetic factors (7).

### 3.1 Lifestyle factors

Over the years, lifestyle factors have been at the core of understanding CVD incidence and risk of premature mortality; they have demonstrated a major role in mediating such diseases and their progression.

Blood cholesterol levels can negatively affect vascular mortality. In fact, one systematic review (SR) and meta-analysis of 61 prospective studies discussed this association (8). Among 150,000 participants with available data on cholesterol levels, a decrease of 1 mmol/L of total cholesterol showed a 56% decrease, 34%, and 17% decrease in ischemic heart disease (IHD) mortality in the age groups of 40–49, 50–69, and 70–89 years, respectively (8). Additionally, blood pressure (BP) has long been studied as a factor of association with CVD mortality; a large meta-analysis of 61 prospective studies accounting for 112.7 million person-years showed that an increase of systolic BP (SBP) of 20 mmHg and an increase of diastolic BP (DBP) of 10 mmHg doubled the risk of IHD mortality in people aged 40–69 years (9). A similar meta-analysis was conducted on 123 randomized control trials (RCTs) which reported a relative risk (RR) of 0.87 (95% CI 0.84; 0.91) of all-cause mortality for every 10 mmHg reduction in SBP (10). Besides elevated cholesterol levels and BP, body mass index (BMI) was linked to a significant increase in all-cause mortality in a meta-analysis that included 239 prospective studies (11). This association is specifically evident for vascular mortality as reported by a collaborative analysis of 57 prospective cohort studies with 894,576 participants with a mean age of 46 (12). The findings of this study indicate that with each 5 kg/m^2^ increase in BMI, there was an average increase in vascular mortality of 40% (95% CI 1.37;1.45) (12). Other factors have also been investigated in the pursuit of understanding CVD mortality, such as smoking and dietary habits. One prospective cohort study looking at the impact of smoking on CVD, with participants’ mean age of 44.4 and 43.6 years in men and women, respectively, found a significant association between being a current smoker and CVD incidence when compared to never-smokers with a reported hazard ratio (HR) of 2.10 (95% CI 1.65; 2.67) (13). Another SR of 18 SR and meta-analysis studies found that cannabis smoking had a higher damaging impact on the cardiovascular system when compared to tobacco (14). Smoking, in addition to other negative behaviors, contributes to what is referred to as a poor lifestyle in terms of health and wellness. Interestingly, over a 20 year follow-up period, a prospective analysis on 88,940 women examining primordial prevention of CVD revealed that 73% of coronary heart diseases (CHD) can be attributed to failure to adhere to a healthy lifestyle, defined as dietary habits that follow the Healthy Eating Index, not smoking, a normal BMI, engaging in physical activity, and limiting alcohol consumption (15).

### 3.2 Genetic factors

There is growing evidence in the literature that genetics plays a significant role in the development of CVD; and that this relationship is further strengthened when it interacts with lifestyle factors. A study that aimed to quantify the genetic risk for coronary artery disease (CAD) in three prospective studies, with a total of 33,296 participants, showed that those who are placed in the high genetic risk quintile contributed to 91% of the incidence of coronary events when compared with those in the low genetic risk quintile (HR 1.91 95% CI 1.75; 2.09) (16). Additionally, this study found that among those ranked in the high risk group, following a healthy lifestyle was associated with a 54% decrease in coronary events (95% CI 0.47;0.63) when compared to those following an unhealthy lifestyle (16).

The various risk factors mentioned can influence the incidence and mortality from CVD in the population, thus the importance of implementing effective prevention strategies at the different stages of disease progression. The subsequent sections will address primary and secondary prevention measures that can effectively manage the risk of premature mortality.

## 4 Primary prevention

Primary prevention aims to prevent the onset of CVD and can be achieved through changes in modifiable factors, which can be mainly categorized as maintaining a healthy diet, smoking cessation, and engaging in physical activity.

### 4.1 Healthy diet

A healthy diet is a key component of primary prevention of CVD. Two diets have been extensively studied in the literature for that purpose; the Dietary Approaches to Stop Hypertension (DASH) and the Mediterranean diet (MD).

The DASH diet emphasizes the consumption of fruits and vegetables, low-fat dairy, nuts, and legumes and was developed as a means to treat hypertension (HTN) (17). An umbrella review of SR and meta-analysis of prospective studies and RCTs investigated the association between a DASH diet and CVD incidence (17). The primary outcome indicated that the DASH diet was associated with a 20% decrease in the incidence of CVD. For example, the study highlighted that DASH dietary patterns significantly decreased SBP and DBP (17). This evidence is further supported by a SR and meta-analysis that included 15 cohort studies and a sample size of 1,020,642 that found a significant association between the DASH diet and a reduction in the risk of all-cause mortality (RR 0.78, 95% CI 0.75; 0.81) and of cardiovascular mortality (RR 0.78 95% CI 0.75; 0.81) (18).

On the other hand, the MD is based on consumption of fruits, vegetables, and legumes with reliance on olive oil as a source of fat, and poultry and fish as a source of protein (19). The adherence to this diet was found to be associated with a decrease in several CVD risk factors. A SR and meta-analysis of 7 prospective cohort studies reported that a strict adherence to the MD was significantly associated with a 9% decrease in the risk of overweight and obesity (95% CI 0.88; 0.94), which, as discussed above, have been tied to higher CVD mortality (20). Another SR and meta-analysis investigated the effect of a MD in RCTs; the findings showed that adhering to the MD decreased SBP by 1.4 mmHg and DBP by 1.5 mmHg on average, in addition to witnessing a 13% decrease in HTN when compared to low adherence (19). By decreasing risk factors, this was accompanied with a decrease in all-cause mortality and CVD mortality as was shown in yet another SR that looked at the results of 6 RCTs (21). It was reported that the MD was linked to 37% reduction in the risk of major cardiovascular events (MACE) (95% CI 0.53; 0.75) (21).

In addition to overall dietary patterns like DASH and Mediterranean diets, specific nutrient and food-group changes offer powerful levers for CVD prevention. The WHO recommends reducing adult sodium intake to <2 g/day (≈5 g/day salt), a target shown to lower blood pressure and reduce risks of stroke and coronary heart disease (22). Moreover, increasing dietary fiber intake (e.g., to ∼25–30 g/day) has been associated with reductions in CVD incidence and improved cardiometabolic risk factor profiles (23). Additionally, replacing portions of saturated fats with polyunsaturated fats yields substantial benefit. Recent studies report that reducing saturated fat intake and replacing it with polyunsaturated fat lowers cardiovascular disease risk (24, 25). Finally, ultra-processed foods and sugar-sweetened beverages are major policy targets: higher intake of added sugars and SSBs increases risk of CVD and stroke; similarly, ultra-processed foods have been linked with higher overall CVD and coronary heart disease risk in prospective cohorts (26, 27).

### 4.2 Smoking cessation

The 2021 ESC guidelines identify smoking as a major causal risk factor for CVD and strongly recommend complete cessation at all ages by emphasizing that all forms of tobacco exposure, including e-cigarettes, increase cardiovascular risk (28) and leads to a 10-year decrease in life expectancy, on average (29). When interpreting the relationship of CVD incidence with smoking in a study of 8,770 individuals with a mean age of 42.2, participants who quit smoking within 5 years compared to current smokers had a significantly lower risk with a reported HR negative difference of 4.51 (95% CI −5.90; −2.77) (30). A SR and meta-analysis of 13 studies of adults between 23 and 53 years reported a decrease of 5.2% m/s in arterial fitness, a risk factor of CVD (29). Another meta-analysis comprising of 25 cohorts in the CHANCES consortium, studied the relationship between smoking status and cardiovascular mortality in 503,905 participants aged 60 and above (31). The main finding of the study was the presence of a dose-response decrease in the risk of cardiovascular mortality with a longer cessation period; the HR for trend per 10-years was 0.85 (95% CI 0.82; 0.89) (31).

A large prospective cohort demonstrated that combustible cigarette smoking significantly increases cardiovascular risk across multiple subtypes, with dose–response gradients (e.g., HR = 2.45 for myocardial infarction; HR = 5.06 for peripheral artery disease) and risk reduction after cessation (32). A systematic review and meta-analysis of 8.5 million participants found that risk from e-cigarettes was primarily concentrated among dual users with combustible tobacco (OR = 2.56), while the specific analysis for exclusive e-cigarette use was not significantly associated with cardiovascular events (33). In parallel, a cross-sectional analysis of over 430,000 adults reported that daily cannabis use was linked to increased odds of myocardial infarction (OR = 1.25) and stroke (OR = 1.42), with stronger associations among never-tobacco users (MI OR = 1.49; stroke OR = 2.16), and clear dose–response by frequency of use (34). Collectively, these findings highlight that combustible tobacco remains the dominant causal driver of cardiovascular disease, while e-cigarette risk is largely mediated by dual use, and cannabis carries independent, dose-related associations even after adjustment for tobacco and e-cigarette use.

### 4.3 Physical activity

Regular physical activity has been well-examined in the literature as an important component of primary prevention of CVD and its risk factors. This was analyzed in a SR of 16 studies looking at the impact of training interventions on cardiovascular risk factors in healthy young and middle-adults; it reported a significant decrease in the participants’ BP and BMI. For instance, reductions in SBP and DBP ranged from 5 to 9 mmHg, and BMI reductions ranged from 1.0 to 1.5 kg/m^2^, both considered clinically significant for reducing cardiovascular event risk (35). A SR and meta-analysis of 16 studies with a minimum of follow-up of two years focused on a more specific intervention, muscle-strengthening, and its impact on mortality from major non-communicable diseases (36). A dose-response decrease in CVD mortality was found with every increase in 10 min/week of muscle-strengthening exercises, with the lowest RR of 0.82 (95% CI 0.76; 0.90) at 60 min/week. When combined with aerobic activities, performing muscle-strengthening exercises was associated with a 46% decrease in the risk of CVD (36). Moreover, a SR and meta-analysis that included 29 prospective studies looking at the association between physical activity and heart failure, found that those who perform high rigorous exercises or high walking intensity compared to lower activity levels had a decrease of 34% and 27% in the risk of heart failure, respectively (37).

Current adult-guidelines recommend 150–300 min/week of moderate-intensity aerobic activity (or 75–150 min/week vigorous, or an equivalent mix) plus muscle-strengthening on ≥2 days/week (38). It further emphasizes that prolonged sedentary time is independently associated with higher risk of all-cause mortality, CVD mortality, type-2 diabetes and adverse cardiometabolic outcomes (38).

### 4.4 Obesity and weight management

Obesity is a major global driver of CVD and contributes to cardiovascular mortality independently of other risk factors (39). Excess adiposity increases the risk of metabolic disorders and accelerates the development of CAD and heart failure. A SR and meta-analysis that included five studies with 881,692 participants found that obesity was significantly associated with an increased risk of CAD with an OR of 1.20 (95% CI 1.02; 1.41) (40). Similarly, a meta-analysis of 38 studies reported that higher BMI in early life was positively associated with cardiovascular outcomes, with a HR of 1.18 (95% CI 1.07–1.30) for CVD, 1.13 (95% CI 1.07–1.19) for CHD, and 1.16 (95% CI 1.11–1.20) for heart failure (41).

Weight loss and weight control interventions have been shown to significantly improve cardiovascular outcomes. An umbrella review of SRs of 31 articles comprising 47 effect sizes found that weight management strategies were associated with reduced all-cause mortality and improved cardiovascular outcomes. Importantly, high- to moderate-quality evidence highlighted the benefits of GLP-1 receptor agonists (GLP-1 RAs) in individuals with type 2 diabetes or overweight, underscoring their role not only in glycemic control but also in weight management and cardiovascular prevention (42).

## 5 Secondary prevention

Secondary prevention is considered an essential part of managing the incidence and premature mortality from CVD. Several strategies can be employed for early detection of CVD in high-risk populations to prevent the progression of the disease.

### 5.1 Screening for CVD

Screening is an important tool of early detection of many burdensome diseases, however, its use to detect CVD to allow early intervention has been a subject of debate with several concerns arising regarding the effectiveness and benefits of this strategy. In fact, a SR of 16 RCTs and cohort studies described the benefits of CVD screening with resting and exercise electrocardiography; the findings highlight that by adding exercise electrocardiography to the risk factors of CVD, such as smoking and cholesterol, there was no significant decrease in cardiovascular events (43). Furthermore, the United States Preventive Service Taskforce recommends screening for blood lipids in men and women before the age of 35 and 45, respectively. The taskforce found inadequate evidence to support the need for CVD screening for men and women below these age thresholds, as well as in children and young adults up to 20 years old (44).

Polygenic risk scores have emerged as powerful tools for cardiovascular risk stratification, summarizing the cumulative effect of numerous genetic variants associated with CAD, and can identify high-risk individuals even before traditional risk factors manifest (45). In parallel, biomarkers such as high-sensitivity C-reactive protein, high-sensitivity troponins, and natriuretic peptides (e.g., NT-proBNP) have shown significant value in detecting subclinical disease and predicting adverse cardiovascular outcomes, potentially enabling earlier initiation of preventive interventions (46–48).

Furthermore, a recent scoping review highlighted how AI algorithms can analyze complex genetic and clinical data to identify individuals at elevated CVD risk, potentially enabling earlier interventions even in patients with subclinical disease (49). AI-based models have also shown strong performance in analyzing electronic health records and imaging data to stratify risk (50–52). However, most of these tools have been validated retrospectively, raising concerns about calibration, transportability across populations, and reliance on surrogate endpoints rather than hard cardiovascular outcomes (52). Bias in training datasets, underrepresentation of marginalized and LMIC populations, and limited regulatory guidance further constrain generalizability and adoption (53).

Ractical applications are emerging, such as AI-enabled ECG analysis and machine learning–enhanced calculators embedded in electronic health records, which may improve risk stratification and resource allocation (51, 52). Nonetheless, large-scale prospective trials are still needed to confirm whether these approaches reduce cardiovascular events. At present, precision medicine and AI should be viewed as complementary to, rather than replacements for, established prevention strategies.

These screening approaches fall under secondary prevention, as they aim to detect risk or subclinical disease in individuals already at elevated risk, rather than preventing the initial development of CVD.

### 5.2 Aspirin therapy

Aspirin is no longer routinely recommended for primary prevention, as guideline bodies highlight that in older adults the bleeding risk often outweighs cardiovascular benefit; use is individualized for select high-risk patients. In contrast, aspirin remains a cornerstone of secondary prevention following myocardial infarction, stroke, or established CVD (28).

Aspirin is an anti-platelet agent that works on reducing the formation of blood clots that can lead to ischemic stroke or heart failure in people with CVD. Many historical trials have tested for the efficacy of aspirin on preventing CVD, starting with the Cardiff I trial from 1974 conducted on 1,239 men who had a recent myocardial infarction episode; this study reported a relative reduction in mortality of 25% (54). A SR looked at the role of aspirin in secondary prevention of CVD; it included 6 primary prevention trials and 16 secondary prevention RCTs with a total of 112,000 low- and high-risk individuals with 660,000 person-years (55). The study reported a decrease of serious vascular events (6.7% vs. 8.2% per year, *p*-value < 0.0001) and a decrease in the number of strokes (4.3% vs. 5.3% per year, *p*-value < 0.0001) (55). These findings were further supported by an RCT exploring the effect of aspirin use in the prevention of first cardiovascular events in diabetes mellitus patients. The study included 15,480 participants; individuals were followed for an average of 7.4 years. The group receiving aspirin experienced a significantly lower incidence of serious vascular events compared to the placebo group (8.5% vs. 9.6%, respectively) with a rate ratio of 0.88 and a 95% CI: 0.79–0.97. However, aspirin use was associated with a higher risk of major bleeding episodes, occurring in 4.1% of participants, compared to 3.2% in the placebo group (rate ratio 1.29; 95% CI: 1.09–1.52). The majority of these bleeding complications were gastrointestinal or other extracranial hemorrhages (56). This trial illustrates the limited role of aspirin in primary prevention, in contrast to its well-established efficacy in secondary prevention.

### 5.3 Statin therapy

Statins are recommended for both primary prevention, among individuals with elevated LDL cholesterol or high estimated ASCVD risk, and for secondary prevention in patients with established CVD, where the benefits are particularly robust; however, their use is primarily focused on secondary prevention (28).

Statins are a class of drugs that work by lowering lipid-blood levels, specifically LDL. This can help prevent further damage to cardiovascular health. A meta-analysis of 28 RCTs examined major vascular events after statin intervention on 183,419 participants with mild kidney disease (57). The findings of this study indicate that following a statin-therapy decreases the risk of MACE by 21% (RR 0.79, 95% CI 0.77; 0.81) per mmol/L reduction in LDL cholesterol (57). These results are further supported by a more recent SR of 6 RCTs looking at the effect of statin on 53,107 participants with CVD or risk factors of CVD; the study reported a 30% reduction in major coronary events, and a 19% reduction in major cerebrovascular events (58).

### 5.4 Non-statin agents

For patients at very high cardiovascular risk who do not achieve LDL-cholesterol goals with statin therapy, or who are statin intolerant, non-statin agents provide additional benefit. Ezetimibe, when added to statins, has been shown to further reduce MACE, particularly in secondary prevention populations with established coronary disease. In the IMPROVE-IT trial, which enrolled 18,144 patients post-acute coronary syndrome, adding ezetimibe to simvastatin lowered median LDL levels to 53.7 mg/dL compared to 69.5 mg/dL with statin alone, and reduced the composite endpoint of cardiovascular death, MI, stroke, revascularization, or unstable angina (HR 0.936, 95% CI 0.89;0.99) (59).

More recently, PCSK9 inhibitors have demonstrated robust LDL-lowering effects and significant reductions in recurrent cardiovascular events in high-risk patients. The FOURIER trial randomized 27,564 patients with ASCVD and LDL ≥ 70 mg/dL on statins to evolocumab, a PCSK9 inhibitor, or placebo (60). Evolocumab reduced LDL cholesterol by 59% (to a median of 30 mg/dL) and lowered the risk of MACE (HR 0.85, 95% CI 0.79; 0.92) and the key secondary outcome of CV death, MI, or stroke (HR 0.80, 95% CI 0.73;0.88) over a median 2.2 years of follow-up (60). These therapies are primarily indicated in secondary prevention, especially in those with persistent LDL elevation despite maximally tolerated statins.

Ezetimibe is generally well tolerated, though mild gastrointestinal symptoms such as abdominal discomfort, diarrhea, or excessive gas may occur, along with some joint pain or respiratory tract symptoms in a small proportion of users (61). Meanwhile, PCSK9 inhibitors have favorable safety profiles overall; the most frequent adverse effects are injection-site reactions, sometimes accompanied by mild flu-like symptoms or general musculoskeletal discomfort. Serious adverse events are rare (62). Additional barriers include cost and mode of administration which may limit widespread use (63, 64).

### 5.5 SGLT2 inhibitors and GLP-1 receptor agonists

Sodium-glucose cotransporter-2 inhibitors (SGLT2i) and GLP-1 RAs are newer therapies with substantial evidence for cardiovascular prevention (65). The strongest evidence for these agents comes from secondary prevention trials in patients with type 2 diabetes and established ASCVD. SGLT2i lower glucose through glycosuria and produce modest reductions in body weight and blood pressure, while also exerting direct cardiovascular benefits (66, 67). GLP-1 RAs improve glycemic control and promote weight loss, with consistent evidence of cardiovascular protection, particularly in patients with type 2 diabetes and obesity (65, 68).

Large cardiovascular outcome trials and meta-analyses have shown that GLP-1 RAs significantly reduce MACE, cardiovascular mortality, stroke, and all-cause mortality across a wide range of patients with and without prior CVD (65, 68, 69). A pooled analysis of 13 CVD outcome trials, including more than 83,000 patients reported reductions in MACE (OR 0.86), cardiovascular mortality (OR 0.87), and stroke (fatal OR 0.74, nonfatal OR 0.87) with GLP-1 RA therapy (68).

SGLT2i have also shown robust and consistent cardioprotective effects. In a large real-world study of over 313,000 patients with type 2 diabetes, SGLT2i use was associated with a 34% lower risk of CVD compared with other second-line therapies (66). A meta-analysis of 13 RCTs involving 90,413 patients found that SGLT2i reduced the composite of first heart failure hospitalization or cardiovascular death by 23%–24% across populations with heart failure, diabetes, or chronic kidney disease, with parallel reductions in cardiovascular mortality (67). Evidence from routine care populations further suggests that SGLT2i may provide greater benefit in patients with a history of heart failure, while GLP-1 RAs may be more effective in reducing atherosclerotic events in those without prior CVD (69).

### 5.6 Antihypertensives

Pharmacological BP reduction remains a cornerstone of both primary and secondary prevention of CVD. A large systematic review and meta-analysis of randomized controlled trials demonstrated that for every 10-mmHg reduction in systolic BP, the relative risk of MACE decreased by about 20%, with consistent benefit across age groups and baseline risk profiles (10). The 2024 ESC guidelines on the management of HTN recommend initiating drug treatment in individuals with established cardiovascular disease if blood pressure is ≥130/80 mmHg despite lifestyle measures, with a treatment target of 120–129/70–79 mmHg if tolerated, while generally avoiding reductions below 120/70 mmHg (70). First-line agents include thiazide-like diuretics, ACE inhibitors or ARBs, and calcium channel blockers, with choice tailored to comorbidities and tolerability (28, 71). These therapies reduce both first cardiovascular events and recurrent events in patients with prior CVD, underscoring their dual role in primary and secondary prevention.

### 5.7 Polypill

The concept of combining multiple cardiovascular medications into a single pill has been tested in large-scale randomized trials. The TIPS-3 trial evaluated a polypill containing simvastatin (40 mg), atenolol (100 mg), hydrochlorothiazide (25 mg), and ramipril (10 mg), with or without low-dose aspirin (75 mg), in 5,713 participants followed for a mean of 4.6 years (72). Compared with placebo, the polypill lowered LDL cholesterol by approximately 19 mg/dL and systolic blood pressure by about 5.8 mmHg (72). The primary outcome of MACE occurred in 4.4% of participants in the polypill group vs. 5.5% in the placebo group (HR 0.79; 95% CI 0.63–1.00) (72). When combined with aspirin, the benefit was enhanced (HR 0.69; 95% CI 0.50–0.97) (72). These findings highlight the synergistic effect of combining lipid-lowering, antihypertensive, and antiplatelet therapy into a single formulation, improving adherence while providing clinically meaningful reductions in cardiovascular risk.

Further management strategies such as cardiac rehabilitation, surgical interventions, and revascularization procedures fall under tertiary prevention. These approaches, while important for reducing morbidity and mortality in advanced diseases, are beyond the scope of this review.

## 6 Discussion

A comprehensive overview of the different risk factors associated with CVD was presented along with potential interventions that could limit premature mortality caused by these diseases. The focus on premature CVD mortality, together with the integration of lifestyle and pharmacological strategies across the full prevention continuum, underscores the relevance of this synthesis. Framing prevention within the SDG 2030 target emphasizes its public health importance, while emerging tools, such as AI-enabled risk prediction and newer cardioprotective medications, illustrate how approaches are evolving beyond traditional strategies.

As with any disease, preventive efforts are multi-leveled. Primary prevention strategies for CVD emphasized adhering to a healthy diet (notably the DASH diet and MD), smoking cessation, and engaging in physical activity, all of which significantly decrease the risk of mortality. At a higher level, secondary prevention strategies for individuals with high-risk CVD highlight the use of different pharmacological therapies in reducing overall risk of CVD mortality. It is important to recognize that all the strategies discussed throughout the review are usually intertwined, and no clear separation is evident. In practice, interventions often offer a synergistic effect in prevention and treatment of CVD, and it is recommended that, even with pharmacological or surgical interventions, patients are still advised and encouraged on the importance of a healthy lifestyle.

AI and machine learning models that integrate clinical, imaging, and genetic data show promise for improving early identification of high-risk individuals and tailoring the intensity of lifestyle counseling and pharmacotherapy. However, most models remain methodologically limited, with challenges in validation, calibration, and generalizability. At present, these tools are best viewed as complementary to guideline-based prevention strategies rather than replacements.

While this review highlights the established role of aspirin and statins in secondary prevention, it also discusses the growing evidence for SGLT2i and GLP-1 RAs and other medications as novel therapies with demonstrated cardiovascular benefits. Their inclusion reflects how prevention strategies are expanding beyond traditional agents, offering additional opportunities to reduce premature CVD mortality when combined with lifestyle interventions.

Despite strong evidence for both lifestyle and pharmacological interventions, translation into clinical practice remains inconsistent. Common challenges include low adherence to lifestyle changes, often influenced by behavioral, cultural, and environmental factors, as well as socioeconomic barriers that restrict access to healthy foods, safe spaces for physical activity, and regular medical care. At the health-system level, resource constraints and persistent disparities between high-income and low- and middle-income countries further hinder equitable implementation of preventive strategies (73–75). Moving forward, efforts may focus on integrated care models that combine patient education, community-based interventions, and access to essential medicines. Additionally, advances in precision medicine and novel pharmacotherapies such as PSCK9 inhibitors provide additional tools but also raise issues of affordability and accessibility.

Emerging opportunities also include digital health interventions, such as telemedicine and mobile-based coaching that may help sustain lifestyle changes, specifically regarding CVD outcomes (76, 77). Those remain understudied across diverse populations and settings, even though studies have highlighted their cost-effectiveness (78). At the policy level, measures like salt reformulation have shown cost-effective cardiovascular benefits, yet their adoption is inconsistent worldwide (79). Importantly, the burden of premature CVD and the feasibility of prevention strategies in low- and middle-income countries remain underrepresented in the literature, highlighting a need for greater equity-focused research (80–82).

This review has some limitations; even though a comprehensive report has been detailed, it is not a SR and hence may have missed some major studies on potential interventions for premature CVD mortality. Moreover, the scope of this review presents a broad overview of possible primary and secondary prevention strategies and sheds light on several interventions that may be useful in preventing premature mortality with little insight on implementation strategies.

## 7 Conclusion

Premature CVD remains a global public health challenge, but a substantial proportion of this burden is preventable. Evidence consistently supports the benefits of lifestyle modification, particularly healthy dietary patterns, smoking cessation, and physical activity, alongside pharmacologic therapies such as statins, antihypertensives, and novel agents like SGLT2i and GLP-1 RAs. Integrating these interventions across the continuum of primary, secondary, and tertiary prevention can substantially reduce early mortality.

Moving forward, greater attention is needed to equity and implementation: ensuring that proven strategies reach diverse populations, especially in low- and middle-income countries, and addressing barriers related to cost, access, and adherence. Emerging opportunities, including artificial intelligence for risk prediction, digital health tools to support behavior change, and population-level policies (e.g., salt reformulation, trans-fat bans), highlight how prevention is evolving. Future research should focus not only on refining risk stratification and expanding therapeutic options, but also on translating evidence into practice through integrated, scalable, and equitable approaches.
